# A smart rotary technique versus conventional pulpectomy for primary teeth: A randomized controlled clinical study

**DOI:** 10.4317/jced.53968

**Published:** 2017-11-01

**Authors:** Negar Mokhtari, Alireza-Sarraf Shirazi, Masoumeh Ebrahimi

**Affiliations:** 1 Pediatric Dentist; 2 Professor of Pediatric Dentistry, Department of Pediatric Dentistry, School of Dentistry, Mashhad University of Medical Sciences, Mashhad, Iran; 3 Dental Materials Research Center, Mashhad University of Medical Sciences, Mashhad, Iran; 4 Associate Professor of Pediatric Dentistry, Department of Pediatric Dentistry, School of Dentistry, Mashhad University of Medical Sciences, Mashhad, Iran; 4 Dental Research Center, Mashhad University of Medical Sciences, Mashhad, Iran

## Abstract

**Background:**

Techniques with adequate accuracy of working length determination along with shorter duration of treatment in pulpectomy procedure seems to be essential in pediatric dentistry. The aim of the present study was to evaluate the accuracy of root canal length measurement with Root ZX II apex locator and rotary system in pulpectomy of primary teeth.

**Material and Methods:**

In this randomized control clinical trial complete pulpectomy was performed on 80 mandibular primary molars in 80, 4-6-year-old children. The study population was randomly divided into case and control groups. In control group conventional pulpectomy was performed and in the case group working length was determined by electronic apex locator Root ZXII and instrumented with Mtwo rotary files. Statistical evaluation was performed using Mann-Whitney and Chi-Square tests (*P*<0.05).

**Results:**

There were no significant differences between electronic apex locator Root ZXII and conventional method in accuracy of root canal length determination. However significantly less time was needed for instrumenting with rotary files (*P*=0.000).

**Conclusions:**

Considering the comparable results in accuracy of root canal length determination and the considerably shorter instrumentation time in Root ZXII apex locator and rotary system, it may be suggested for pulpectomy in primary molar teeth.

** Key words:**Rotary technique, conventional technique, pulpectomy, primary teeth.

## Introduction

One of the most important purposes of pediatric dentistry is to maintain deciduous teeth for functional and esthetic purposes, preserve arch length and normal eruption of permanent teeth. In spite of a decreased rate of caries prevalence in many countries , deep caries in deciduous teeth making pulp therapy inevitable in most children (1).

Accurate determination of working length to ensure complete removal of pulpal tissue is one key factor in success rate of pulpectomy treatments (2). On the other hand, decreasing the duration of procedure is necessary for appropriate behavior management of children.

A clinical study by Saritha *et al.* (3), on accuracy of root canal length measurement in deciduous teeth with Root ZX II apex locator showed favorable results .In another study conducted by Neena *et al.* (4), the accuracy of root canal measurement in both techniques, apex locator and digital radiography were comparable with paralleling technique. The results of an ex vivo study by Beltrame *et al.* (5), proved that there was no statistically significant difference between the accuracy of determining working length with Root ZX apex locator and conventional technique. An *in-vivo* study by Beltrame *et al.* (5), on comparing the accuracy of the two different apex locators, Root ZX and Ipex , showed no significant difference in results compared with conventional radiography. The same results were found in a similar study performed in–vitro by Nelson-Filho *et al.* (6). Mello-Moura *et al.* (7), conducted an *in-vitro* study to compare Root ZX apex locator and conventional radiography with a direct observation technique and found that the accuracy of the apex locator technique was superior to conventional radiographs.

Several studies showed quality of root canal filling is significantly better in Mwo rotary system than conventional method (8-10). In an *in-vivo* study performed by Makarem *et al.* (11), on comparing the the quality of canal filling of rotary and manual instrumentation in mandibular second primary molars, similar results were found between the both techniques.

Considering the fact that most of the studies in this area have been performed in-vitro, further *in-vivo* studies on the advantages of the combination of these two methods are recommended.

The aim of the present study was to determine the accuracy of root canal length measurement with Root ZX II apex locator and rotary system in pulpectomy of primary teeth.

## Material and Methods

This randomized control clinical trial was performed on 80 first or second mandibular primary molars in 80 children aged 4 to 6 year referring to Pediatric Dentistry Department, Mashhad Dental School, Mashhad, Iran. This research has been accepted by the research council and the ethical committee of Mashhad University of Medical Sciences (research code: 900729). Also informed consent was taken from children’s parents. Oral and radiographies examinations have been taken for all children. Then children with mandibular primary molars with indication of pulpectomy treatment, were selected.

Radiographs were obtained by MINRAY®X-ray unit, (Soredex, Tuusula, Finland), with exposure settings of 70kvp, 7mA, 0.25 seconds, vertical angle of -5 degrees and horizontal angle perpendicular to facial surface of tooth structure.

As root resorption can affect the accuracy of apex locator readings, teeth with abscess or root resorption or those with immature roots were excluded from the study.

Patients were randomly divided into equal groups of rotary-apex locator and conventional method. In this study, block randomization technique with the block size of 40 was used. The participants were allocated to groups A or B through random allocation sequence. Both the patients and the result analyzers were blind to the method of treatment; however, the operator (NM)cannot be blind to the method of treatment.

In the conventional method, working length was determined using an endodontic gauge to measure the length between mesial cusp tip up to 2mm to root apex. The same approach was taken for the distal root. Filing was performed using #15 to #35 k-files.

In the rotary-apex locator technique, working length was determined using the Root ZX II apex locator (J. Morita Corp., Tokyo, Japan) and Mtwo rotary files #10, #15 and #20 were utilized for instrumentation. The Rotational speed and torque of the files were adjusted according to the recommendations of the manufacturer.

After instrumentation was over, canals were dried with paper cones and obturated with zinc oxide eugenol paste using a #25 lentulo spiral in a slow speed handpiece . Crown restoration was performed using stainless steel crowns (3M ESPE, St. Paul, MN, USA).

The duration of instrumentation was measured in minutes and seconds. The accuracy of working length measurement was determined by two blinded observers (ME,AS) and was graded as 1, 2 and 3 as follows:

1: Overfilled or an underfilled of more than 4 mm 

2: 2-4mm underfilled from the radiographic apex

3: Adequate filling (less than 2mm from the radiographic apex)

SPSS software version 16 was used for statistical analysis. To describe data, frequency tables and charts were used. The collected data was evaluated with Chi-square and Mann-Whitney tests. *P*<0.05 was set as statistically significant.

## Results

In this study, 80 mandibular primary molars (44 first molar and 36 second molar) of 4-6year-old patients (mean 4.8±0.69) were selected for complete pulpectomy technique. 40 cases were treated with conventional technique and 40 with Root ZX II apex locator and rotary technique. The average instrumentation time was 7.80±1.96 minutes for the conventional method and 4.13±1.51 minutes for the rotary technique and the difference was statistically significant according to t-test analysis (*P*=0.000). The mean ranks for the quality of instrumentation was 41.19 and 39.81 for the conventional and rotary techniques, respectively, so no significant difference was observed (*P*=0.787).

The quality of root canal filling did not have a statistically significant difference between the first and second primary molars (*P*=0.507).  Table 1 shows that the average quality of canal instrumentation did not have a statistically significant difference between the mesial or distal canals in the first and second mandibular primary molars, regarding instrumentation technique ( Table 1).

**Table 1:**
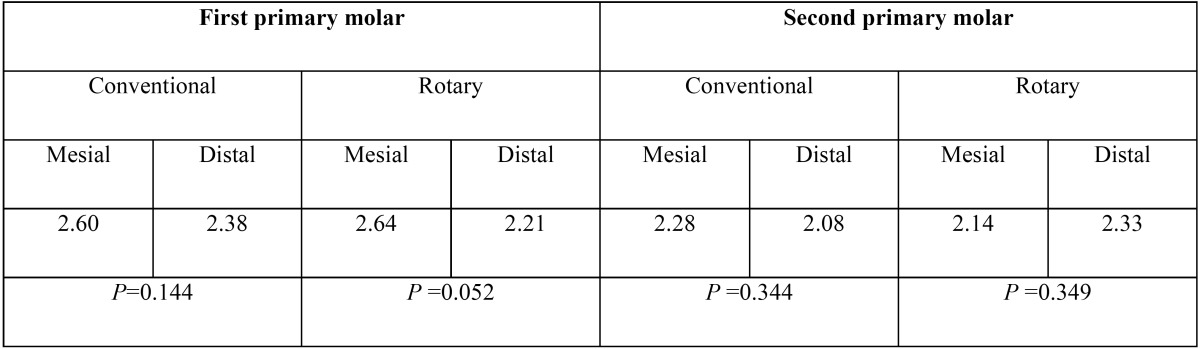
Mean quality of canal filling in mandibular molars according to canals.

There was no significant difference between the quality of root canal filling in the both instrumentation techniques in the first primary mandibular molar (*P*=1) and second primary mandibular molar (*P*=0.082). Also  Table 2 presents frequency distribution of root canal filling quality in primary molars according to the method of instrumentation (*P*=0.940).

**Table 2:**
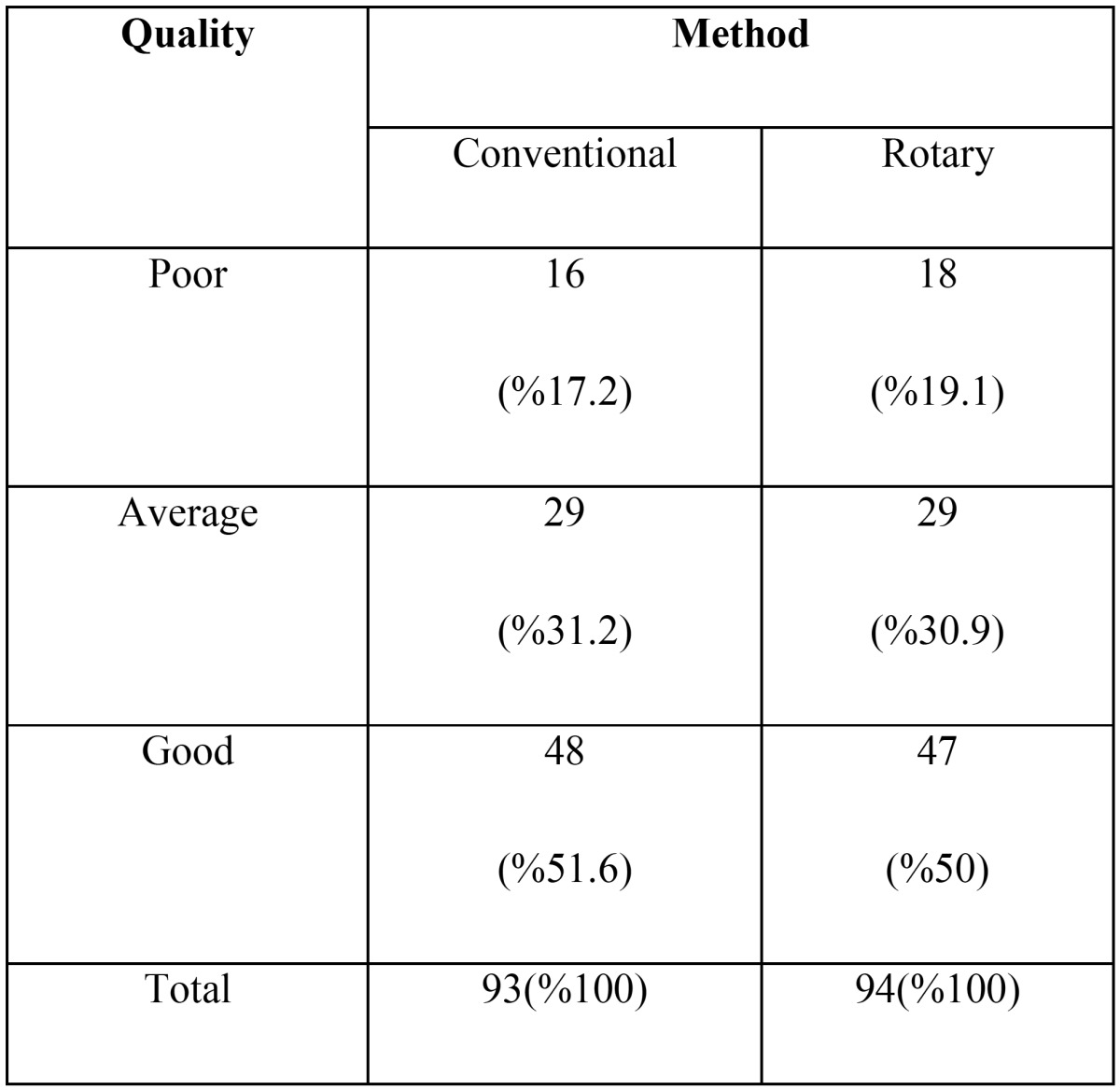
Absolute frequency of root canal filling quality according to instrumentation technique in mandibular primary molars.

## Discussion

The aim of the present study was to compare the effect of conventional versus rotary instrumentation, on the quality and duration of pulpectomy treatment in mandibular primary molars. Also we compared the accuracy of root canal working length determination between apex locator technique and conventional radiography. According to the results of this study, the difference between the mean accuracy of working length was not statistically significant between both techniques (*P*>0.05); however, the duration of instrumentation was considerably lower in the rotary technique compared with conventional technique (*P*=0.000).

A number of studies have been compared the accuracy of electronic apex locators versus conventional radiographic method in primary teeth. The several in vitro studies and an *in-vivo* study by Saritha *et al.* (3-7,12-14), support the results of present study. Using apex locator in root canal length determination of primary teeth seems justifiable which will lead to decreased X-ray exposure and elimination of radiographic examination with subsequent shortening of treatment duration.

In an *in-vivo* study performed by Makarem *et al.* (11), on comparing the quality of root filling of rotary and manual instrumentation in mandibular second primary molars, similar results were found between both techniques; however, the quality of rotary instrumentation was more favorable for the mesial canals. In the present study, the mean accuracy of root canal length determination showed no significant difference between mesial and distal canals. One explanation for the difference in findings could be dissimilarity of the rotary systems utilized. As explained earlier in the present study, Mtwo rotary files and Root ZX II apex locator were used while in Makarem *et al.* study NSK rotary system with Flex master rotary files were used.

Most of the previous studies in this issue have been performed *in vitro*; however, present study was a randomized control clinical trial, which gives our study a privilege compared to similar researches as this method is one of the most valuable methods of investigating clinical interventions.

Also in the present study, the accuracy of root canal length measurement for each tooth type was investigated according to the method of instrumentation. According to the results obtained, the mean accuracy of root canal length measurement in either of the first or second primary molars did not have a meaningful difference between the both methods of measurement.

Due to impossibility of determining the actual position of apical foramens, which is even more variable in primary teeth, the use of electronic apex locators can decrease inaccuracy in measurement compared with radiograph.

Root canals in primary teeth are narrower necessitating the use of smaller files for initiation of filing. One reason for choosing Mtwo rotary files was the accessibility of #10 files in this rotary system.

As it is not possible to determine the apical foramen in primary teeth radiographically due to asymmetric resorption patterns, determining root canal foramen by the qualitative method used in our study can be effective in reducing errors. In this study, the quality of root canal preparation was assessed based on a qualitative ordinal scale, while the previous studies assessed measuring quantitatively in millimetres from the apical foramen.

In the present study, the mean time for canal preparation was 4.13±1.51 minutes in rotary instrumentation and 7.80±1.94 minutes in conventional method which showed a statistically significant difference (*P*<0.001).

Same finding presented by Garcia-Godoy *et al.* (9), Makarem *et al.* (11), Ochoa-Romero *et al.* (15), Pinheiro *et al.* (16), Kummer *et al.* (17), Azar *et al.* (18) and Ramazani *et al.* (19), that showed the average duration of canal preparation was significantly lower in rotary systems compared with the conventional method.

The reason of choosing rotary and apex locator systems which are combined together in Root ZX II device is to obtain comparable results in root canal length measurement along with decreased treatment duration.

Through a widespread research in databases, it seems that present study is the first randomized control clinical trial that used a system that simultaneously investigate the rotary filing and electronic apex locator in primary molars. One limitation of this system is the higher cost and a more complicated procedure compared with conventional method.

Considering comparable results in determining the length of root canals in both methods along with shorter treatment duration in rotary filing system, the electronic apex locator and rotary instrumentation could be a good option for pulpectomy treatment of primary teeth.
